# Fire Retardancy, Water Absorption, and Viscoelasticity of Borated Wood—Polycarbonate Biocomposites

**DOI:** 10.3390/polym13142234

**Published:** 2021-07-07

**Authors:** Jingfa Zhang, Ahmed Koubaa, Dan Xing, François Godard, Peng Li, Yubo Tao, Xiang-Ming Wang, Haigang Wang

**Affiliations:** 1 Laboratoire de Biomatériaux, Université du Québec en Abitibi-Témiscamingue, Rouyn-Noranda, QC J9X 5E4, Canada; zjf2010dl@126.com (J.Z.); Francois.Godard@uqat.ca (F.G.); 2 State Key Laboratory of Biobased Material and Green Papermaking, Qilu University of Technology, Shandong Academy of Sciences, Jinan 250353, China; lipeng@qlu.edu.cn (P.L.); taoyubo@qlu.edu.cn (Y.T.); 3 New Construction Materials Group, FPInnovations, Québec, QC G1V 4C7, Canada; Wang@fpinnovations.ca; 4 Key Laboratory of Bio-Based Materials Science and Technology (Ministry of Education), Northeast Forestry University, Harbin 150040, China; hgwang@nefu.edu.cn

**Keywords:** polycarbonate, boric-acid treatment, wood flour, biocomposites, fire retardancy, dimensional stability, creep behavior

## Abstract

Demand for high-performance biocomposites is increasing due to their ease of processing, low environmental impact, and in-service performance. This study investigated the effect of boric acid modification of wood flour on polycarbonate (PC) wood composites’ thermal stability, fire retardancy, water absorption, and creep behavior. The composites’ fire retardancy increased with increasing wood flour content, and their char residue increased by 102.3% compared to that of pure PC. However, the water absorption of the resulting composites increased due to the hydroxyl groups of the wood flour. Wood flour also improved the composites’ anti-creep properties. The excellent fire retardancy and anti-creep properties of wood–PC composites expand their use in the construction sector.

## 1. Introduction

Nowadays, wood–polymer composites (WPCs) as a potential material have captured both researchers’ and manufacturers’ attention due to their advanced characteristics, such as rot resistance, durability, low cost, dimensional stability, high stiffness, and strength [1,2,3]. They are widely used in buildings, decoration, transport, furniture, and decking areas [4]. WPC is expanding rapidly since its emergence, and its production is increasing [5]. However, both lignocellulose and polyolefin are flammable. Hence, improving the fire retardancy of biocomposites to expand their utilization to other sectors.

Ammonium polyphosphate (APP) [6,7,8], metal hydroxides [9,10], and intumescent flame retardant [11,12] are among the approaches used to improve the fire retardancy of biocomposites. The common fire-retardant mechanisms of WPCs are inherited from the plastic matrix; that is, the nature of the plastic matrix plays an important role in the fire retardancy of biocomposites. Moreover, composites’ strength decreases with increasing fire-retardant content due to their hygroscopicity and poor compatibility [13,14,15]. Hence, it is important to improve the properties of the matrix for the performance of biocomposites.

Interestingly, polycarbonate (PC) has advanced transparency characteristics, dimensional stability, fire resistance, high heat distortion temperature, outstanding impact resistance, and low creep [16,17]. Combining PC and boric-acid-treated wood flour resulted in biocomposites with improved thermal stability [18]. The PC biocomposites showed outstanding mechanical properties comparable to wood and engineered wood products, such as oriented strand board (OSB) and plywood [18]. Cellulose has been filled into PC to prepare biocomposites based on engineering plastic [19]. PC combined with cellulose produced highly improved composites (100% improvement) compared to neat PC [20]. Advantages of wood fibers include low cost, biodegradability, renewability, and low density, making wood–PC composites environmentally friendly [21,22,23]. Moreover, lignocellulosic fibers improved the biocomposites’ fire retardancy compared to neat polymer [24]. Increasing wood flour content improved the biocomposites’ flame retardancy. Ignition time, peak heat release rate, and rate of burning decreased, whereas the duration of burning increased [25]. Based on the above findings, wood flour/PC composites are suitable for applications where fire retardancy is essential.

However, lignocellulosic fibers are hydrophilic, increasing the water absorption of the resulting biocomposites. Generally, the mechanical properties of biocomposites decrease after water uptake because water molecules change the structure and properties of fibers, the matrix, and the interface between fibers and polymers [2,26]. Water molecules damage the crystalline structure of lignocellulosic fibers and the polymer chain reorientation. The water uptake process can also affect shrinkage. When the fiber–polymer matrix interface is exposed to moisture, the fibers tend to swell after water absorption, resulting in shear stress development at interfaces, leading to their debonding. The water absorption of polyolefin-based biocomposites increases with the increase in lignocellulosic fiber content [27]. Furthermore, water absorption is closely related to biocomposites’ durability and weather resistance [28,29]. Thus, the investigation of water absorption is essential for biocomposites.

Zhang et al. [18] found that wood flour reacted with PC chains through esterification, different from the polyolefin-based WPCs. Esterification will not be observed if the PC is replaced by polyolefin. The compatibility between wood and PC is better than that of wood and polyolefin due to the polar functional groups of PC. Wood flour is evenly distributed in the PC content due to similar polarities [18]. Moreover, a high processing temperature would impact the properties of wood flour, further changing the properties of the composites. This indicates that the structure of wood flour/PC composites is different from that of wood flour/polyolefin composites. Based on the above analysis, it can be summarized that the difference between wood flour/PC composites and wood flour/polyolefin composites is not simply changing the polymer matrix. Thus, it is necessary to explore the fire retardancy and water absorption of the wood flour/PC composites.

Hence, this study explored the effect of borated wood flour content on the fire retardancy and water absorption properties of wood flour/PC composites to promote their application. The fire retardancy of borated wood flour/PC composites was investigated. The microstructure of char layers of wood flour/PC composites explained their fire-retardant mechanism.

## 2. Materials and Methods

### 2.1. Materials

Polycarbonate particles (Makrolon 6485, supplied by Polyone Co., Avon Lake, OH, USA), with a melt flow index of 10 g∙10 min^−1^ (according to ISO 1133, 300 °C, 1.2 kg) and a density of 1.2 g∙cm^−3^ were used. The pure PC has a fire-retardant grade of UL 94 V0. Wood flour (30–80 mesh) was ground from poplar (*Populus tremuloides* L.) veneers provided by LVL Global, an industrial wood mill in northeast Quebec, Canada. Sigma-Aldrich (Oakville, Canada) provided the boric acid, which is an analytical grade reagent.

### 2.2. Preparation of Wood–PC Biocomposites

Wood flour was treated using a boric acid solution of 5 wt% by soaking for 2 h. Then, the wood flour was filtered and dried to a moisture content of 2%. The boric acid treatment successfully improved the thermal stability of wood flour [30]. The PC particles were dried at 100 °C for 10 h in a laboratory oven. The PC particles and the treated wood flour were extruded at 210 °C using a HAAKE PolyLab OS Rheodrive (Thermo Electron (Karlsruhe) GmbH, Karlsruhe, Germany) at a speed of 50 rpm. The wood flour content varied from 0 to 40 wt%, as shown in Table 1. Test samples for mechanical and water absorption were produced using an injection molding machine (MARS II 130/600, Haitian, China). The injection temperature varied from 230 to 245 °C, and the mold temperature was 90 °C. The injection and holding pressures were set at 140 and 70 MPa, respectively. The preparation of wood flour/PC composites without boric acid treatment failed due to the degradation of wood flour. Samples for the cone calorimeter (CONE) test were prepared using a hydraulic laboratory press (Fontijne Presses b.v., Vlaardingen, The Netherlands) at a temperature of 230 °C, and a pressure of 20 MPa held for 4 min. Then, samples were cooled down under the same pressure to room temperature.

### 2.3. Characterization

The fire retardancy of the resulting composites was characterized using a cone calorimeter and a vertical burning test. The cone calorimeter tests were carried out according to the ISO 5660-1-2002 standard with a heat flux of 50 kW·m^−2^. The cone test measures the heat release rate (HRR), the total heat release (THR), the smoke production rate (SPR), and the total smoke production (TSP). Three replicate samples were tested and averaged for each formulation. Furthermore, a vertical burning test was carried out according to the UL94 standard. Test samples of 80 × 10 × 3.2 mm^3^ were suspended vertically and ignited with a gas flame for 10s, and the total burning time (from ignition to extinction) was recorded. Finally, a KEYENCE 3D microscope (VK-X150K, KEYENCE, Osaka, Japan) was used to analyze the microscopic structure of the composites charring residues after the vertical burning test.

The water absorption test was conducted according to the ASTM D 570 standard. Samples with a diameter of 50 mm and a thickness of 3.2 mm were dried at 50 °C for 24 h and then placed in distilled water at room temperature. Dimensions were measured using a micrometer with an accuracy of ±0.001 mm, while weights were measured using a laboratory balance with an accuracy of ±0.001 g at different intervals up to 720 h. We used six replicates for each formulation and measured the thickness at five parallel points for each sample. Furthermore, the two-hour boiling water immersion test was also carried out according to the ASTM D 570 standard. Six replicates with five parallel measurement points were used for each formulation in the boiling water immersion test.

The creep behavior of the wood flour/PC composites was characterized using a thermomechanical analysis instrument (Q400, TA Instruments, New Castle, DE, USA) with a three-point bending mold under a nitrogen atmosphere. Specimens with dimensions of 13 × 5 × 1 mm were loaded at a constant force of 0.1 N for 600 s at 50 °C.

The resulting composites’ creep curves were fitted by the Burgers and Findley’s models to analyze their viscoelasticity using the Origin 2017 (OriginPro 2017, OriginLab, Northampton, MA, USA) software. The Burgers model, consisting of one Maxwell element and one Kelvin–Voigt element connected in series, is described by Equation (1) [31]:

(1) $$\varepsilon =\frac{\sigma }{E_{1}}+\frac{\sigma }{E_{2}}\left(1-\exp\left(-t\frac{E_{2}}{\eta_{2}}\right)\right)+t\frac{\sigma }{\eta_{1}}$$

where *ε* is the total strain accumulated after the time *t* after the stress *σ* is applied. *E*_1_ and *E*_2_ represent the modulus of elasticity of the springs in the Maxwell and Kelvin–Voigt units, respectively. *η*_1_ and *η*_2_ are the corresponding damping viscosities. Equation (2) describes Findley’s model:

(2) $$\varepsilon \left(t\right)=\varepsilon_{0}+At^{n}$$

where *ε*(*t*) is the time-dependent creep strain, while *ε*_0_ is the instantaneous elastic strain. *A* is the magnitude of the transient creep strain, and *n* is a constant.

## 3. Results and Discussion

### 3.1. Fire Retardancy

**Figure 1:**
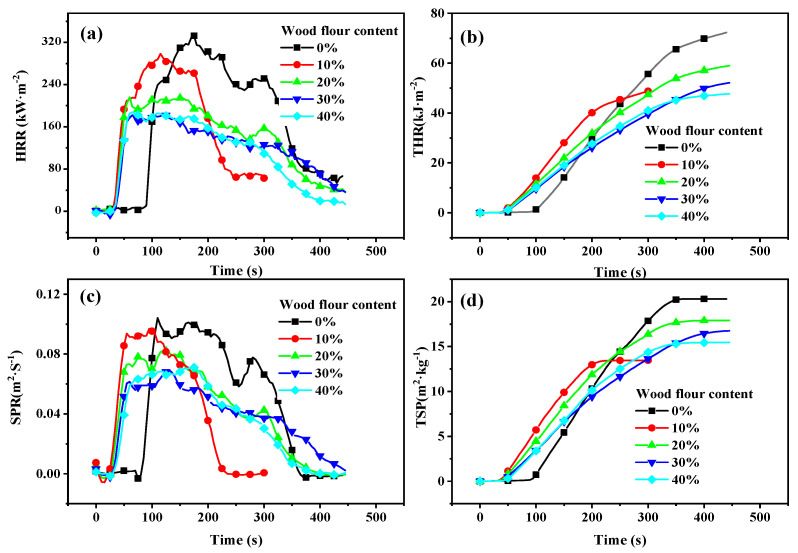
Heat rate release (HRR) (**a**), total heat release (THR); (**b**), smoke production rate (SPR); (**c**), total smoke production (TSP); (**d**), and curves of wood-PC composites.

**Figure 2:**
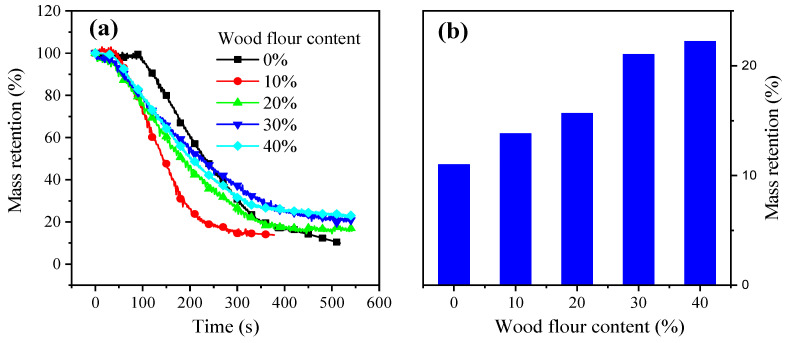
Mass retention curves of wood flour/PC composites (**a**) and the mass residue of the resulting composites at 500 s (**b**).

Figure 1 and Figure 2 and Table 2 show the fire retardancy parameters of the wood–PC composites. These parameters include HRR, the peak of HRR (PHRR), total heat release, total smoke release, ignition time (IT), mass loss rate (MLR), and flaming dripping. IT (Table 2), HRR (Figure 1), and PHRR (Table 2) decreased with increasing wood flour proportion. Compared to PC, the lower thermal stability of wood flour can explain this result [18,19]. The changes in IT were rather marginal, which is consistent with the previous study [32]. The effect of wood flour content on IT is not obvious. Although a limiting oxygen index (LOI) test may help differentiate the effect of wood content, the previous literature cautioned against using the LOI to measure the flammability of WPC. In a real fire situation, factors such as lower oxygen accessibility and higher air velocity and temperature can influence the LOI of the composites [33]. The HRR of the resulting composites decreased gradually with increasing wood flour content (Figure 1b). The PRHH of composites fell from 332.3 kW m^−2^ for neat PC to 187.9 kW m^−2^ for WPC40. The heat release rate decreased by 43.5%. The THR also reduced with the increase in wood flour content. The previous literature reported similar findings [34,35]. The decrease in heat release rate can be explained by the fact that the burning heat of wood flour is lower than that of polymers due to its high oxygen element content [36]. The slope of the THR curve is representative of flame spread. Hence, adding wood flour decreased the flame spread of the resulting composites, as shown in Figure 1b. Similar to HRR, the SPR gradually reduced with increasing wood flour content (Figure 1c), and the TSP curves in Figure 1d show similar results. The degradation of wood flour produced char layers, playing a crucial role in smoke suppression [37].

Moreover, the mass fraction of residue of the resulting composites quickly decreased with the irradiation time after ignition. After approximately 400 s, the mass retention curves tended to level off. However, the combustion of composites with 10% wood flour content was short. HRR and SPR curves show similar trends. This may be due to the fact that the wood flour content is too low to form a protective char layer, and the thermal stability of the wood flour is lower than PC. The residues rate at 500 s increased by 102.27% from 10.99% of neat PC to 22.23% of WPC40 (Figure 2). Mass retention can be attributed to the suppressing effect on the flame combustion, accumulating more char during the combustion. Moreover, the vertical burning test did not show any flaming dripping for all tested samples during combustion. Additionally, a self-extinguishing phenomenon was observed for all samples (Table 2). Neat PC is self-extinguishing, whereas wood flour is the opposite [36]. In summary, wood flour and PC have a synergistic effect on fire retardancy in the wood–PC composites.

### 3.2. Morphological Characterization

**Figure 3:**
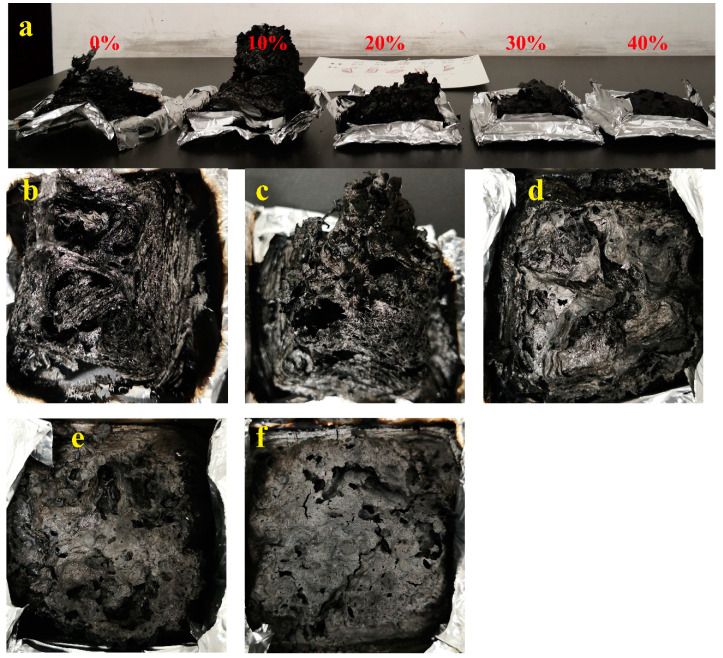
(**a**) Digital photos of char formation for wood–PC composites after cone calorimeter and the surface morphology of char formed from the resulting composites with different wood contents: (**b**) 0%; (**c**) 10%; (**d**) 20%, (**e**) 30% and (**f**) 40%.

The digital photos of wood–PC composites, neat PC, and WPC10 showed evident inflation after the CONE test. This is because PC degradation produced a large amount of carbon dioxide (CO_2_) [38]. Meanwhile, the vaporized CO_2_ could also dilute the flammable gases and oxygen during burning, acting as gas-phase fire retardancy. Hence, the pure PC is self-extinguishing after the removal of the fire resource. However, the inflation of the char residue decreased when the content of wood flour was more than 20%. Additionally, the surface of the residual carbon became flat (Figure 3). Wood flour degraded and formed char layers, suppressing the overflow of volatile gases and burning the composites. Furthermore, the foamed char reduced the thermal conductivity at the material interior, enhancing the fire retardancy of wood flour/PC composite, which was in accord with the above CONE analysis results.

**Figure 4:**
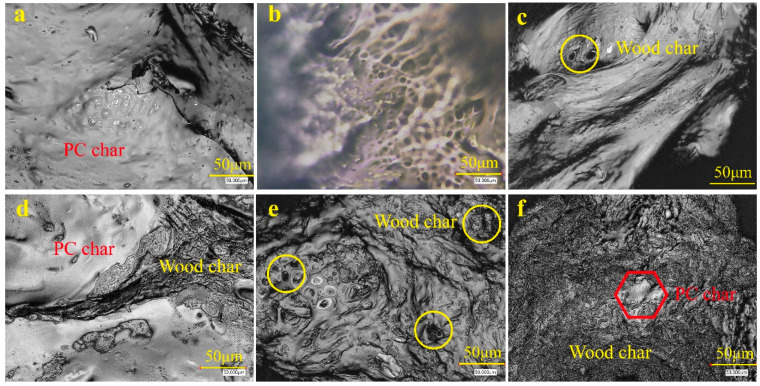
Micromorphology of char formation for wood–PC composites with different wood flour contents after vertical burning test: (**a**,**b**) 0%, (**c**) 10%, (**d**) 20%, (**e**) 30%, and (**f**) 40%.

The morphology of the char layers measured by a laser microscope revealed that the char of pure PC seemed smooth and dense (Figure 4a). A uniform porous carbon framework of char after combustion was also observed (Figure 4b). With the addition of wood flour, an inhomogeneous and relatively loose structure with many flaws appeared on the surface of the char layer. Wood flour formed this loose char structure. Hence, the biochar can prevent heat and flammable gases from penetrating the PC. The biochar increased with increasing wood flour content in composites (Figure 4).

### 3.3. Water Absorption

**Figure 5:**
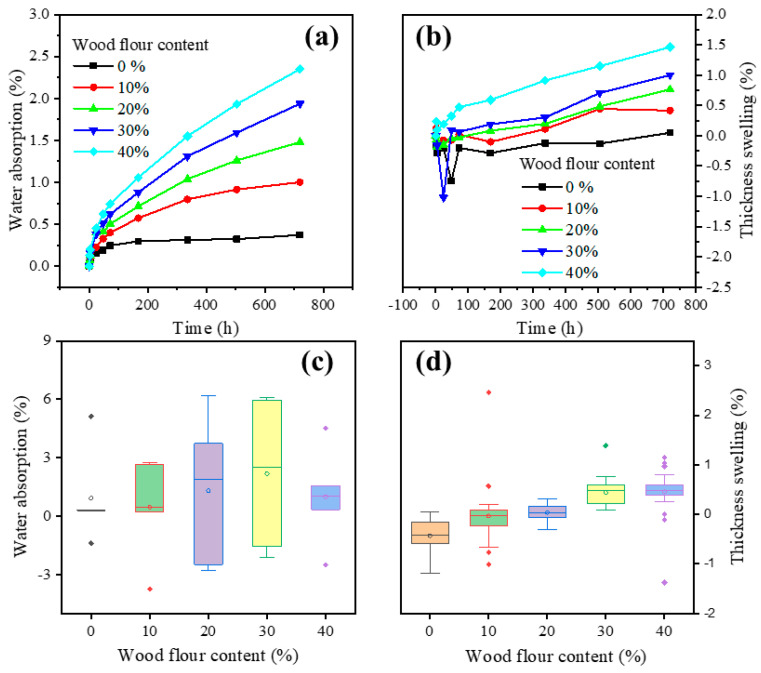
Water absorption (**a**) and thickness swelling (**b**) of wood-PC composites at room temperature, and water absorption (**c**) and thickness swelling (**d**) in two-hour boiling water test.

Figure 5 shows that the water absorption and the thickness swelling of the wood flour/PC composites increased with time and increased wood flour content. The water absorption of composites with 40% wood flour content is about 5.3 times more than pure PC. The wood flour has many free hydroxyl groups on its surface, leading to water absorption [39]. However, the hydrophobic PC matrix is considered the nonabsorbent part of the biocomposites. When immersing the biocomposites in water, the free hydroxyl groups of wood flour formed hydrogen bonds with water molecules [40]. An increasing number of hydroxyl groups of wood flour appeared on the surface of the biocomposites with increasing wood flour content, increasing water uptake. The previous literature reported a similar phenomenon for both wood polypropylene composites and wood HDPE composites [41,42]. For the two-hour boiling water test, the water absorption of the biocomposites did not change obviously with increasing wood flour content. This indicates the excellent hydrothermal stability of the composites. Moreover, the trend of the thickness swelling of the biocomposites in the boiling water test was similar to that in the water absorption test at room temperature. Upon immersion, wood flour on the surface of composites uptakes water and swell; then, the water is transferred from one cell to another over time [39].

### 3.4. Creep Properties

**Figure 6:**
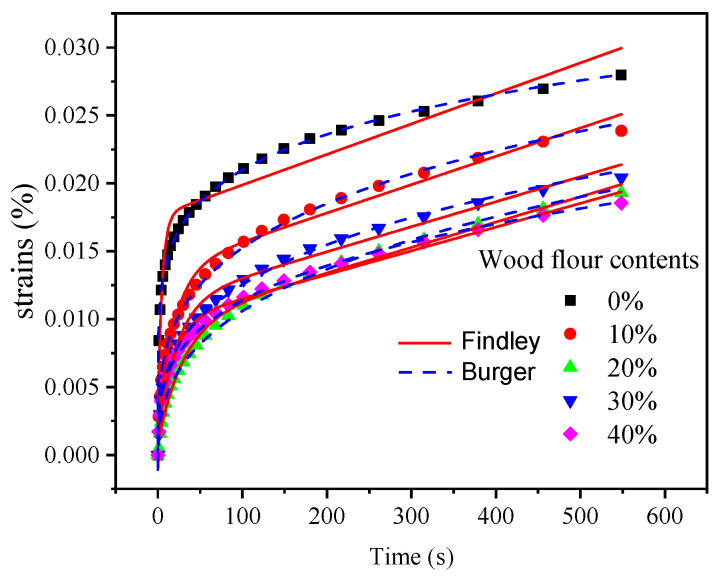
Creep behavior of wood–PC composites.

Creep is an important property for composites since it is indicative of the potential structural applications. Wood content, polymer nature, wood particle size, test time, temperature, applied stress, and humidity influence the creep behavior of biocomposites. Generally, the creep behavior is more severe for biocomposites than for wood due to the nature of polymers [43]. In the current study, the creep strain of the wood–PC composites decreased with higher wood flour content (Figure 6), in good agreement with previous findings for wood–polypropylene composites [44]. The reason for this may be that wood flour restricted the stretching, bending, and slipping of PC molecular chains, which is in good agreement with the modulus results of the composites [18]. Furthermore, the rigidity of wood flour is higher than that of pure PC plastic, which increases the anti-creep property of the resulting wood flour-PC composites [40,45].

Creep occurs due to the viscoelastic deformation of the biocomposites, including elastic deformation and viscous flow. Hence, the Burgers and Findley’s models analyzed the composites’ viscoelastic (creep) performance [30]. Compared to the Burgers model, Findley’s power law model produced a better fit to the data, as shown in Figure 6 and Table 3. Generally, the Burgers model describes the entire creep process well, including instantaneous deformation, viscoelastic deformation, and viscous deformation. The viscoelastic deformation usually occurs in the early stage of creep behavior caused by molecular chain relaxations and extensions. In contrast, the viscous part is the long-term creep strain attributed to the slippage of molecular chains [46]. However, the resulting composites did not contain all three phases for the short-term creep test in this study, especially not the total viscoelastic stage. This explains why the Burgers model did not entirely fit the short-term experimental data. Findley’s power law model fitted the experimental data well. It does not clearly distinguish the two parts of the second stage and does not represent well the viscosity stage. Therefore, it may be more suitable to fit only short-term creep data. The results were in good agreement with the previous literature [43,47].

## 4. Conclusions

In this study, we prepared wood flour/PC composites using injection molding by improving the thermal stability of the wood flour with boric acid and explored the effect of wood flour content on their fire retardancy, water absorption, and creep properties. The results indicate that the wood flour/PC composites had good fire retardancy and were self-extinguishing. Furthermore, the cone calorimeter test parameters, namely, HRR, THR, SPR, and TSP, decreased with increasing wood flour content. However, the water absorption of the composites increased with an increase in wood flour content. Moreover, the creep of the resulting composites decreased with increasing wood flour content. Adding wood flour reduced the cost of PC composites. It improved the fire retardancy and anti-creep properties of the resulting composites. Expanding the use field of biocomposites and promoting their technological development are among the practical implications of this study.

## Figures and Tables

**Table 1:** Formulations of the wood flour-PC composites.

Composition	WPCC0	WPCC10	WPCC20	WPCC30	WPCC40
Wood flour (wt. %)	0	10	20	30	40
Polycarbonate (wt. %)	100	90	80	70	60

**Table 2:** Combustion parameters of wood flour/PC composites.

Samples	IT (s)	PHRR (kW m^−^^2^)	Flaming Dripping	Self-Extinguishing
Neat PC	88	332.3	No	Yes
WPC10	28	298.3	No	Yes
WPC20	31	216.3	No	Yes
WPC30	31	182.8	No	Yes
WPC40	32	187.9	No	Yes

**Table 3:** Parameters of Burgers model and Findley’s power law model.

	Burgers Model				Findley’s Power Law Model				
Wood Flour Content	E_1_ (MPa)	E_2_ (MPa)	η_1_ (MPa s)	η_2_ (MPa s)	R^2^	ε_0_	A	*n*	R^2^
0%	6391.482	1378.851	890,473.587	6951.743	1.00	−6.4998 × 10^−4^	0.00336	0.27717	0.995
10%	8158.469	1787.585	958,608.082	36,341.878	1.00	−1.09 × 10^−3^	0.00248	0.33628	0.995
20%	11,716.722	2089.627	1,086,001.610	48,037.217	1.00	−9.1809 × 10^−4^	0.00363	0.2847	0.995
30%	21,637.338	2286.305	1,070,591.423	70,117.161	1.00	−8.4842 × 10^−4^	0.00474	0.26555	0.996
40%	13,531.938	2410.191	1,142,779.012	43,057.373	1.00	−2.2283 × 10^−4^	0.00977	0.16832	0.998

## Data Availability

The data presented in this study are available on request from the corresponding author. The data are not publicly available because they are still being used for modeling work.
